# Acute Effects of Different Plyometric and Strength Exercises on Balance Performance in Youth Weightlifters

**DOI:** 10.3389/fphys.2021.716981

**Published:** 2021-09-14

**Authors:** Hanen Werfelli, Raouf Hammami, Mohamed Amine Selmi, Walid Selmi, Goran Gabrilo, Cain C. T. Clark, Michael Duncan, Damir Sekulic, Urs Granacher, Haithem Rebai

**Affiliations:** ^1^Research Laboratory: Education, Motor Skills, Sports and Health (EM2S, UR15JS01), Higher Institute of Sport and Physical Education of Sfax, University of Sfax, Sfax, Tunisia; ^2^Higher Institute of Sport and Physical Education of Ksar Said, Manouba University, Tunis, Tunisia; ^3^Tunisian Research Laboratory “Sports Performance Optimization”, National Center of Medicine and Science in Sports (CNMSS), Tunis, Tunisia; ^4^Faculty of Kinesiology, University of Split, Split, Croatia; ^5^Centre for Intelligent Healthcare, Coventry University, Coventry, United Kingdom; ^6^Centre for Sport, Exercise and Life Sciences, Coventry University, Coventry, United Kingdom; ^7^Division of Training and Movement Sciences, University of Potsdam, Potsdam, Germany

**Keywords:** postural stability, conditioning exercise, adolescents, performance, weightlifting

## Abstract

**Background:** High-intensity muscle actions have the potential to temporarily improve the performance which has been denoted as postactivation performance enhancement.

**Objectives:** This study determined the acute effects of different stretch-shortening (fast vs. low) and strength (dynamic vs. isometric) exercises executed during one training session on subsequent balance performance in youth weightlifters.

**Materials and Methods:** Sixteen male and female young weightlifters, aged 11.3±0.6years, performed four strength exercise conditions in randomized order, including dynamic strength (DYN; 3 sets of 3 repetitions of 10 RM) and isometric strength exercises (ISOM; 3 sets of maintaining 3s of 10 RM of back-squat), as well as fast (FSSC; 3 sets of 3 repetitions of 20-cm drop-jumps) and slow (SSSC; 3 sets of 3 hurdle jumps over a 20-cm obstacle) stretch-shortening cycle protocols. Balance performance was tested before and after each of the four exercise conditions in bipedal stance on an unstable surface (i.e., BOSU ball with flat side facing up) using two dependent variables, i.e., center of pressure surface area (CoP SA) and velocity (CoP V).

**Results:** There was a significant effect of time on CoP SA and CoP V [*F*(1,60)=54.37, *d*=1.88, *p*<0.0001; F(1,60)=9.07, *d*=0.77, *p*=0.003]. In addition, a statistically significant effect of condition on CoP SA and CoP V [*F*(3,60)=11.81, *d*=1.53, *p*<0.0001; F(3,60)=7.36, *d*=1.21, *p*=0.0003] was observed. Statistically significant condition-by-time interactions were found for the balance parameters CoP SA (*p*<0.003, *d*=0.54) and CoP V (*p*<0.002, *d*=0.70). Specific to contrast analysis, all specified hypotheses were tested and demonstrated that FSSC yielded significantly greater improvements than all other conditions in CoP SA and CoP V [*p*<0.0001 (*d*=1.55); *p*=0.0004 (*d*=1.19), respectively]. In addition, FSSC yielded significantly greater improvements compared with the two conditions for both balance parameters [*p*<0.0001 (*d*=2.03); *p*<0.0001 (*d*=1.45)].

**Conclusion:** Fast stretch-shortening cycle exercises appear to be more effective to improve short-term balance performance in young weightlifters. Due to the importance of balance for overall competitive achievement in weightlifting, it is recommended that young weightlifters implement dynamic plyometric exercises in the fast stretch-shortening cycle during the warm-up to improve their balance performance.

## Introduction

Weightlifting is a sport that involves lifting the heaviest possible weight during two events: the snatch, and the clean and jerk lifts. Both lifts are powerful movements requiring the ability to produce high levels of muscle strength and power (Pearson et al., 2002; Stone et al., 2005) while maintaining balance over a small base of support, i.e., feet (Riemann et al., 2020). For instance, weightlifting involves tremendous power production and motor-skill coordination throughout the body which may prompt unique sensory information acquisition and integration adaptations (Riemann et al., 2020). Given that the task of weightlifting is associated with high-velocity perturbations to stability and maintaining balance (Bryanton and Bilodeau, 2018), weightlifters likely experience adaptations in acquiring and integrating sensory information, as well as motor strategies, for maintaining balance. Especially, for young weightlifters, balance capacity plays a critical role in their performance due to the need to control heavy weights above head height for at least a couple of seconds with their arms in fully extended position (Kang et al., 2013). In this context, investigators involved in this sport recommend to implement balance training in weightlifters’ training routines to improve balance performance which is a prerequisite for sport-specific performance (Kang et al., 2013). Such observations are directly supported by studies which evidenced balance as a fundamental motor skill directly related to the proper development of weightlifting performance (Kang et al., 2013; Szafraniec et al., 2020).

Different studies have indicated that, when applied as single intervention, ballistic (high velocity) strength training (Pancar et al., 2017), plyometric (Twist et al., 2008), and balance (Hammami et al., 2021) training have the potential to improve balance performance. For balance training, this can be expected with reference to the principle of training specificity (Behm and Sale, 1993). Recently, the reported transfer effects from ballistic strength and plyometric training to balance performance are evident. Of note, this finding is relatively robust because many studies were able to replicate effects of ballistic strength or plyometric training on balance performance in different cohorts (Granacher et al., 2011; Asadi et al., 2015). While the training effects of these regimens are similar on a performance level, they differ substantially with respect to their neural control mechanisms (Taube et al., 2008; Lauber et al., 2021).

Recent long-term studies using either eccentric (Hammami et al., 2020) or traditional strength training or Olympic weightlifting programs (Chaouachi et al., 2014) have proven to be effective to enhance balance performance in youth weightlifters. In this context, Chaouachi et al. (2014) showed significant increases for measures of static balance (i.e., stork test) following 12weeks of traditional strength and weightlifting training, whereas Hammami et al. (2021) showed no improvement in dynamic balance (i.e., Y-balance test) in prepubertal weightlifters following 6weeks of hamstring eccentric training. However, there is no study available that examined whether the reported facilitating effects observed with long-term balance training also exist after a single bout of strength training in youth. Plyometric training is a dynamic form of strength training involving either fast (i.e., drop jump) or slow (i.e., countermovement jump) stretch-shortening cycle actions during the performance of vertical and horizontal jumps and displacements of the center of gravity (Komi and Bosco, 1978; Taube et al., 2006). Flanagan et al. (2007) showed that the precise mechanisms which underpin any given slow or fast stretch-shortening cycle may be determined by the demands of the respective stretch-shortening cycle criterion task. For example, during the fast stretch-shortening cycle, the plyometric exercise (e.g., drop jump) is performed in a highly dynamic mode with short ground contact time which affords high levels of postural control to successfully perform the exercise (Komi et al., 1987; Kibele et al., 2014). Moreover, according to the concept of training specificity (Behm and Sale, 1993), the dynamic nature of the exercises during the fast stretch-shortening cycle would place a training stress on postural control or equilibrium. The increased negative speed during a fast compared to a slow stretch-shortening cycle increases the speed of prestretch of the knee extensors and the plantar flexors and decreases the delay between the prestretch and the concentric phase (Bobbert et al., 1987). This again may lead to a greater mechanical output during the push-off phase which can improve the sensitivity of afferent feedback pathways (Borghuis et al., 2008) leading to faster onset times of stabilizing muscles (Anderson and Behm, 2005). Thus, fast stretch-shortening cycle exercises seem to meet these requirements of being specific and inducing training-specific responses. Hence, it can be postulated that among the different types of strength training exercises, plyometric exercises particularly conducted in the fast stretch-shortening cycle best mimic the demands of the subsequent balance task (Bobbert et al., 1987; Komi et al., 1987; Kibele et al., 2014; Lauber et al., 2021). While the chronic effects of ballistic strength, plyometric and balance training are well-documented on balance performance (Twist et al., 2008; Granacher et al., 2011; Hammami et al., 2016), and less is known about the acute effects of the different types of strength exercises on subsequent balance performance.

Therefore, the aim of this study was to determine the acute effects of different types of strength exercises including plyometrics in fast (FSSC) and slow (SSSC) stretch-shortening cycle exercises, dynamic (DYN) and isometric (ISOM) strength exercises performed during one session on subsequent balance [center of pressure surface area (CoP SA) and velocity (CoP V)] performance in young weightlifters. With reference to the relevant literature (Twist et al., 2008; Haycraft et al., 2016; Pancar et al., 2017), we hypothesized that the implementation of plyometric and dynamic strength exercises would lead to acute performance enhancements in measures of balance in youth weightlifters. Given that the performance of plyometrics particularly in FSSC affords high levels of postural control to successfully perform the exercise (Bobbert et al., 1987; Komi et al., 1987; Kibele et al., 2014; Lauber et al., 2021), we speculated that FSSC would elicit the greatest changes in subsequent balance performance in youth athletes.

## Materials and Methods

### Participants

Overall, 16 young weightlifters (5 girls and 11 boys) were enrolled in this study to make sure that we would not fall short in terms of study population due to sickness or injuries. All participating athletes were members of a Tunisian weightlifting performance center (Table 1). Participants were involved in systematic weightlifting training for at least 2.4years. At the time of this study, they trained 2–3 times per week with 90min per session. It is important to note that all athletes had regularly performed back squats and specific weightlifting exercises (i.e., snatch, and clean and jerk) during competitions and training for a minimum of 1year before the start of the study. Data recorded for this study were taken during training. Legal guardians and participants provided informed consent and assent after a thorough explanation of the objectives and scope of the research project, including the procedures, risks, and benefits of the study. The study was conducted according to the latest version of the Declaration of Helsinki, and the protocol was fully approved by the Local Ethics Committee of the National Centre of Medicine and Science of Sports of Tunis (CNMSS-LR09SEP01) before the commencement of the assessments. None of the participating athletes had a history of musculoskeletal, neurological, or orthopaedic disorders that might have impaired their ability to execute the prescribed strength exercise protocols and balance test.

**Table 1:** Participants’ anthropometric and dynamic strength characteristics in the whole sample, male and female young weightlifters.

	Whole sample (*n*=16)	Males (*n*=11)	Females (*n*=5)
Age (years)	11.3 ± 0.6	11.4 ± 0.8	11.5 ± 0.5
Training experience (years)	2.4 ± 0.8	2.3 ± 0.8	3 ± 1
Body height (cm)	148.1 ± 6.4	147.3 ± 7.9	152.4 ± 5.5
Body mass (kg)	36.5 ± 6.9	36.5 ± 9.4	38.7 ± 7.5
Sitting height (cm)	62 ± 20.5	66.6 ± 21.1	63.8 ± 31.3
Leg length (cm)	86.1 ± 18.9	80.7 ± 18.6	88.6 ± 33.8
Body fat (%)	6.6 ± 1.3	6.3 ± 1.2	7.6 ± 2.1
Peak height velocity	−1.9 ± 1	−2.5 ± 0.6	−0.4 ± 0.3
APHV	13.3 ± 0.8	14 ± 0.6	11.9 ± 0.6
1 RM (kg)	40.5 ± 12.6	42.5 ± 14.1	39 ± 16.7

*Values are means and standard deviations (SD); APHV, age at peak height velocity; and 1 RM, one repetition maximum*.

#### Sample Size

With reference to the study of Pancar et al. (2017) on the acute effect of anaerobic exercise (i.e., Wingate power test) on dynamic balance, an *a priori* power analysis, with a type I error rate of 0.05 and 80% statistical power, was computed. The analysis indicated that 10 participants would be sufficient to observe significant, large effects (Cohen’s d=0.8) for dynamic balance performance (i.e., anteroposterior displacement of the CoP).

#### Procedures

The testing was conducted in an indoor weightlifting performance center. One week before the commencement of the study, all athletes participated in an orientation session to become familiar with the strength exercise protocols and the balance test. A certified strength and conditioning specialist instructed the young weightlifters on how to perform the balance test. Participants were also tested on submaximal 10 maximum repetition (RM) back squat performance in order to define the individual loads which were later applied in the strength protocols. This method has previously been shown to be a valid tool for evaluating strength changes by relatively high (RM) values (Faigenbaum et al., 2007; Faigenbaum, 2009), and the procedure avoids safety-related issues of performing 1RM testing with a pediatric population. Each athlete’s body height and mass were collected using a wall-mounted stadiometer (i.e., OHAUS, Florhman Park, NJ, United States) and an electronic scale (i.e., Baty International, West Sussex, England), respectively. The sum of skinfolds was assessed using the Harpenden skinfold calipers. Body measurements were conducted according to Deurenberg et al. (1990) who reported similar prediction errors between adults and adolescents. Pubertal timing was estimated according to the biological age of maturity for each gender, as described by Moore et al. (2015). After the warm-up, participants completed a baseline (pre-intervention) balance test. Thereafter, the athletes performed in randomized order the four strength exercise protocols (SSSC, FSSC, ISOM, and DYN) on consecutive days with at least 48h in between the different experimental conditions. After the strength exercise protocol, all weightlifters participated in post-intervention balance test. The applied strength protocols were randomly performed after the warm-up and were part of the participating athletes’ regular weigthlifting training regimens.

#### Balance Performance

Balance performance was evaluated in bipedal stance with eyes opened on an unstable surface (i.e., BOSU ball with the flat side facing up) using a force plate with three strain gauges and a sampling rate of 40Hz (PostureWin©, Techno Concept^®^, Cereste, France). The bipedal position was selected because the strength exercises were also performed in bipedal mode. To increase balance difficulty and to avoid a ceiling effect, the BOSU ball was put on top of the force plate. Participants were asked to stand as still as possible during testing with their arms comfortably placed downward at either side of the body; their bare feet were separated by an angle of 30° and their heels placed 5cm apart. To maintain the same foot position for the balance assessment, a plastic device was used that allowed replication of the foot position. Throughout testing, participants were instructed to look straight ahead at a cross, placed at eye level on a nearby wall (2m distance). Each test trial lasted 30s. As dependent variables, two CoP sway parameters were analyzed (i.e., CoP SA in mm^2^ and velocity in mm/ms). More specifically, CoP V indicates the total distances covered by the CoP divided by the duration of the sampled period and CoP SA represents the ellipse of the area covered by the trajectory of the CoP (Schubert and Kirchner, 2014). For these parameters, the lower the value, the better the balance performance (Caron et al., 2000). Intraclass correlation coefficients (ICCs) are presented in Table 2 for the respective CoP parameters.

**Table 2:** Test–retest reliability of variables.

	ICC3.1 (95% CI)	SEM	CV (%)
CoP SA (mm2)	0.86 (0.61–0.95)	1.17	0.53
CoP V (mm/ms)	0.91 (0.74–0.96)	2.66	0.38

*ICC, intraclass correlation coefficient; SEM, standard error of measurements; CV (%), coefficient of variation percent; CoP SA, center of pressure surface area; CoP V, center of pressure velocity*.

#### Protocols of the Applied Strength Exercises

**Figure 1:**
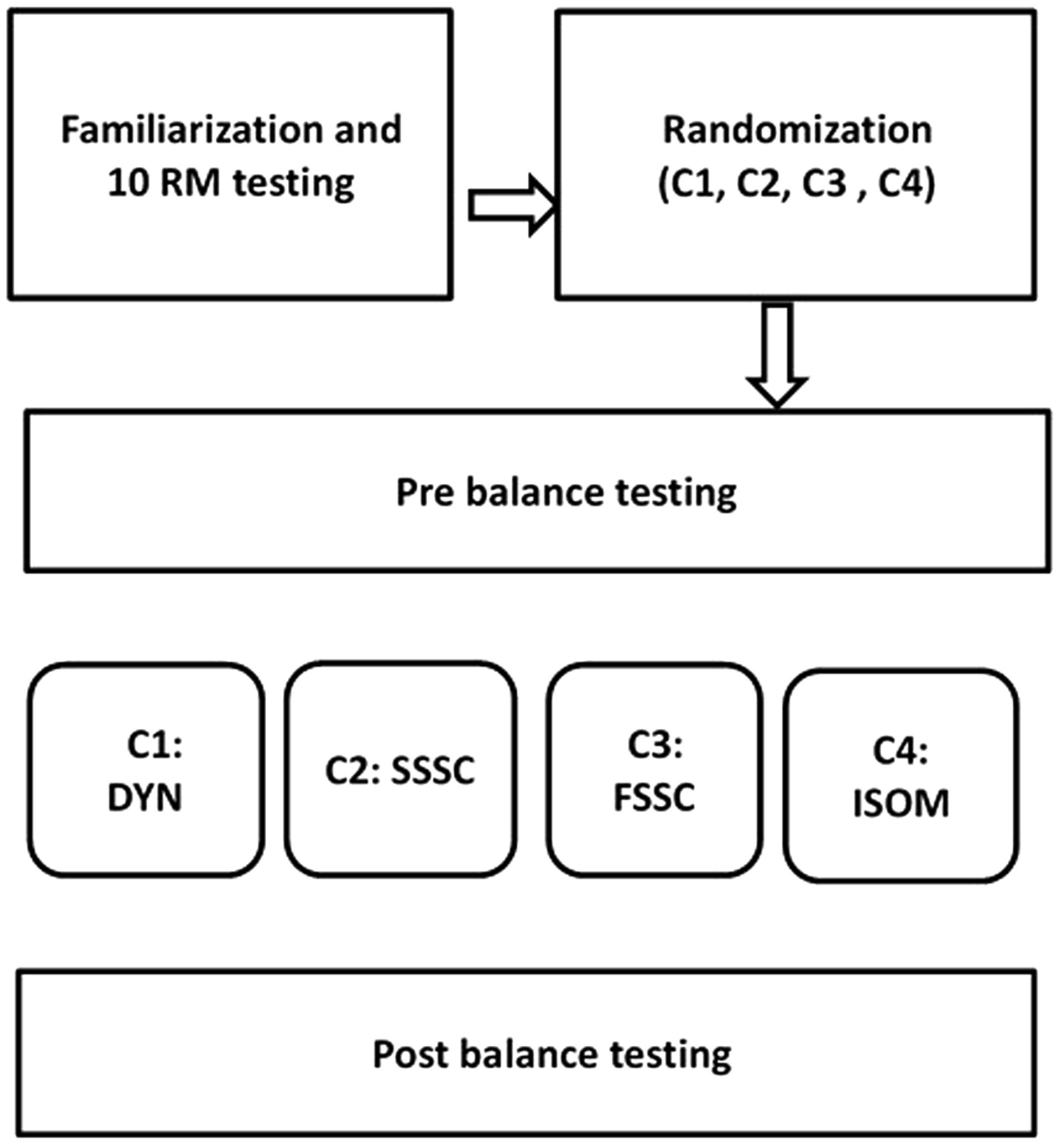
Experimental design and study execution. DYN, dynamic strength exercise; ISOM, isometric strength exercise; SSSC, slow stretch-shortening cycle exercise; FSSC, fast stretch-shortening cycle exercise; 10 RM, 10 repetition maximum.

Each strength exercise protocol lasted 20min, including 10-min warm-up, and consisted of four strength exercises, namely, SSSC, FSSC, ISOM, and DYN (Figure 1). The SSSC comprised 3 sets of 3 hurdle jumps over 20cm hurdles. The FSSC consisted of 3 sets and 3 repetitions of drop jumps from a drop height of 20cm. The DYN protocol comprised back squats with 3 sets of 3 repetitions at a load of the 10 RM. The ISOM consisted of 3 sets of 3-s voluntary contractions while performing the back squat at a knee angle of 90° with a load corresponding to the 10 RM. The strength exercises were performed on a smith machine to ensure that the bar could not begin moving during both ISOM and DYN protocols. Because fatigue can influence the performance of explosive movements and possibly increase the risk of injury (Faigenbaum et al., 2006) and based on previous strength training studies in youth (Sander et al., 2013), rest intervals between sets during each protocols comprised 3min to allow for adequate recovery. Each session began with a standardized 10-min warm-up, including submaximal intensity running, dynamic stretching, calisthenics, and preparatory exercises (e.g., squatting and jumping exercises at a progressively increased intensity). Participants were also instructed to refrain from any strenuous activities before the test sessions. To minimize confounding factors, instructions related to sleep and diet were given to all athletes before the experiment started.

## Statistical Analyses

Data are presented as means and standard deviations (SD), and normality was assessed and confirmed using the Shapiro–Wilk test. The data were then analyzed using a 4 (condition: SSSC, FSSC, ISOM, and DYN) by 2 (time: pre, post) ANOVA for repeated measures. Where the assumption of sphericity was violated, Greenhouse–Geisser correction was used to interpret the results. Where any significant differences were found, post-hoc pairwise comparisons (Tukey) were used. Additionally, effect sizes (ES) were determined from ANOVA by converting partial eta-squared to Cohen’s d, in accord with Cohen (1988). Moreover, within-group ES were computed using the following equation: ES=(mean post – mean pre)/SD. In accordance with Hopkins (2002), ES were considered to be either “trivial” (<0.2) “small” (>0.2–0.6), “moderate” (>0.6–1.2), “large” (>1.2–2), or “very large” (>2). Test re-test reliability of the variables was assessed using Cronbach’s model of ICCs and standard error of measurements (SEM) according to the method of Hopkins (2005).

For the varying conditions, i.e., SSSC, FSSC, ISOM, and DYN, contrast analyses (Haans, 2008; Hervé and Williams, 2010) were carried out to specifically test the following hypotheses; (H1) FSSC would lead to greater changes in the CoP measures than all other conditions (SSSC, DYN, and ISOM; H2) FSSC would yield greater improvements in the CoP measures than DYN and ISOM, and (H3) FSSC would yield greater improvements in the CoP measures than SSSC. Accordingly, three contrasts were computed. First, we compared the FSSC condition vs. DYN, ISOM, and SSSC conditions. The second analysis compared the FSSC condition vs. DYN and ISOM. Finally, the third contrast compared the FSSC condition vs. the SSSC condition. This approach yielded a comparison of one (or more) condition(s) vs. the grand mean of the specified contrasts. Indeed, post-hoc analyses, while useful, do not yield sufficient insight into multiple levels or detailing patterns in response, whereas contrast analysis allows researchers to test theory-driven expectations directly against empirically derived group or condition (Rosnow and Rosenthal, 1995, 1996). The level of significance was set at *p*<0.05. The statistical analysis was carried out using IBM SPSS (version 25).

## Results

All 16 weightlifters completed the study according to the study design and methodology. Participants attended all testing sessions, and none reported any exercise- or test-related injury. Table 2 displays the test–retest reliability analyses for all the balance test parameters. ICCs showed good reliability for all balance parameters and ranged from 0.86 to 0.91, with a SEM from 5.77 to 6.16. Furthermore, a paired t-test showed no significant differences between the scores recorded during the two trials for all measured variables.

### ANOVA and Post-hoc Analyses

There was a statistically significant main effect of time for CoP SA and CoP V [*F*(1,60)=54.37, *d*=1.88, *p*<0.0001; F(1,60)=9.07, *d*=0.77, *p*=0.003]. In addition, there was a statistically significant main effect of condition for CoP SA and CoP V [*F*(3,60)=11.81, *d*=1.53, *p*<0.0001; F(3,60)=7.36, *d*=1.21, *p*=0.003]. Moreover, significant condition-by-time interactions were found for CoP SA (*p*<0.003, *d* =0.54) and CoP V (*p*<0.002, *d*=0.70; Table 3).

**Table 3:** Results of the two-way ANOVA for repeated measures.

Variables	Conditions	Pre-intervention	Post-intervention	ANOVA value of p (Cohen’s d)		
				Time	Condition	Condition x Time
CoP SA (mm2)	DYN	1313.7	1363.1	0.0001 (1.88)	0.0001 (1.53)	0.003 (0.54)
	ISOM	1278.7	1074.1			
	FSSC	1388.8	831.7			
	SSSC	1470.6	1250.6			
CoP V (mm/ms)	DYN	67.7	62.2	0.003 (0.77)	0.003 (1.21)	0.002 (0.70)
	ISOM	64.1	63.0			
	FSSC	68.4	55.5			
	SSSC	65.2	64.8			

*Values are expressed as means and standard deviations (SD), CI 95% confidence limits; DYN, dynamic strength protocol; ISOM, isometric strength protocol; SSSC, slow stretch-shortening cycle; FSSC, fast stretch-shortening cycle, CoP SA, center of pressure surface area; CoP V, center of pressure velocity.*

Between-condition post-hoc testing highlighted statistically significant differences for FSSC between DYN and ISOM [*p*<0.0001 (*d*=1.01); *p*<0.0001 (*d*=0.87), respectively], for CoP SA. There were also statistically significant differences for FSSC between DYN and ISOM [*p*<0.0001 (d=0.80); *p*<0.0001 (*d*=0.65), respectively], for CoP V. Within-group post-hoc tests showed that for CoP SA, both DYN and ISOM were significantly lower post-intervention. DYN and ISOM resulted in significantly lower CoP V, and FSSC in significantly higher CoP V, post-intervention.

### Contrast Analyses

**Figure 2:**
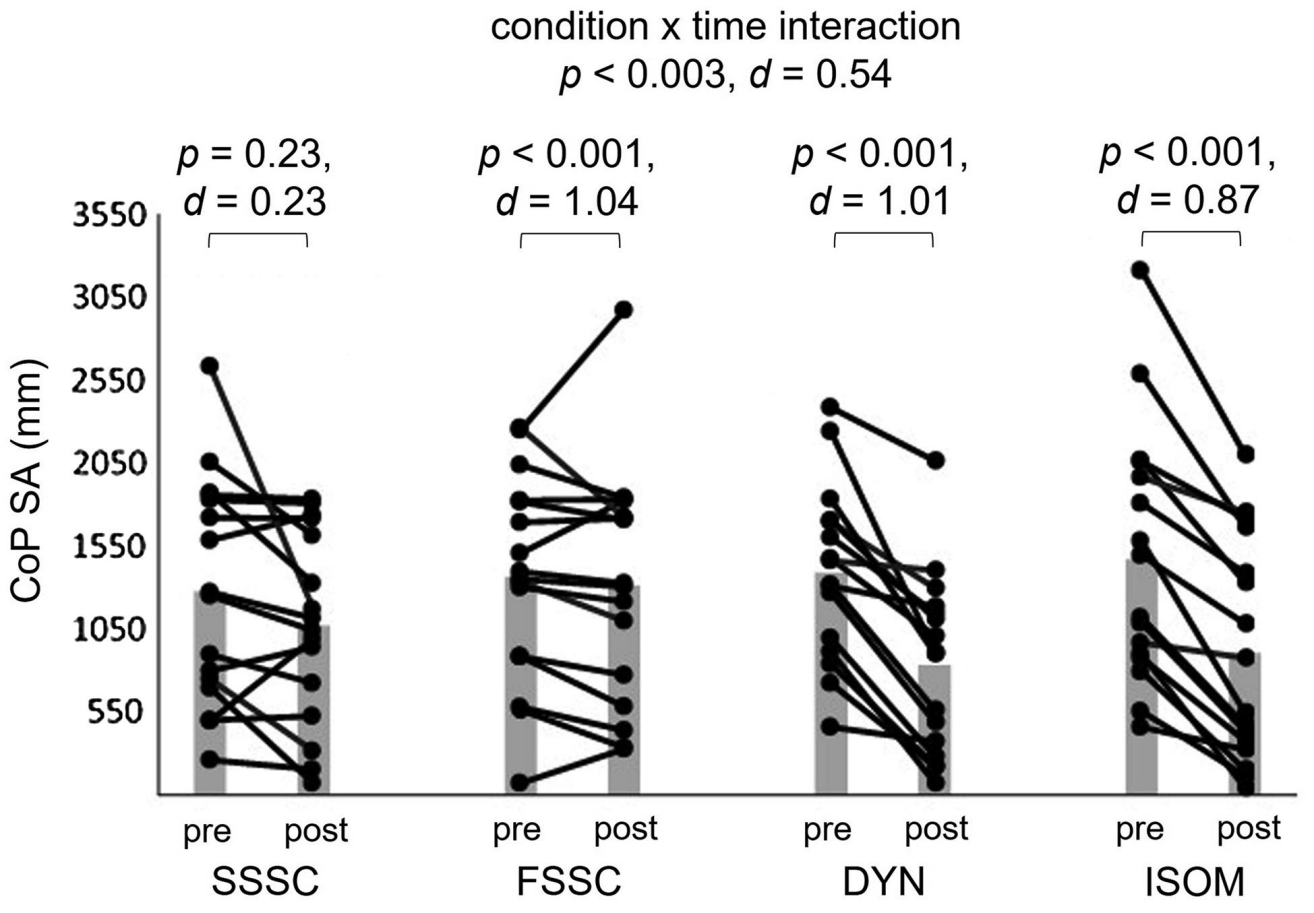
Mean value (grey bar) and individual scores of the center of pressure surface area pre- and post-intervention. DYN, dynamic strength exercise; ISOM, isometric strength exercise; SSSC, slow stretch-shortening cycle exercise; FSSC, fast stretch-shortening cycle exercise; CoP SA, center of pressure surface area; d, Cohen’s d.

**Figure 3:**
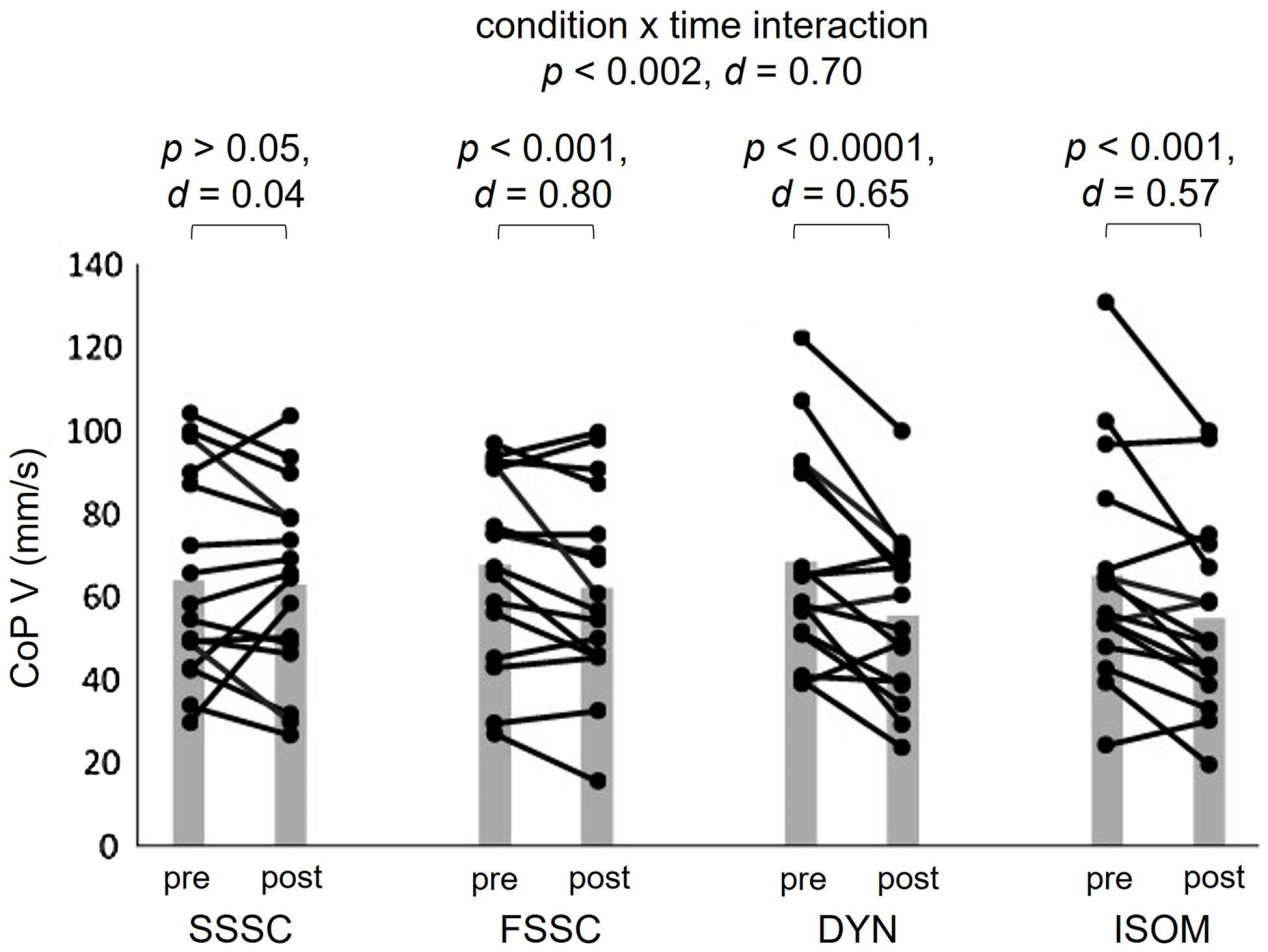
Mean value (grey bar) and individual scores of the center of pressure velocity pre- and post-intervention. DYN, dynamic strength exercise; ISOM, isometric strength exercise; SSSC, slow stretch-shortening cycle exercise; FSSC, fast stretch-shortening cycle exercise; CoP V, center of pressure velocity; d, Cohen’s d.

Specific to contrast analyses, all specified hypotheses were tested and are detailed in Table 4 and Figures 2, 3. For contrast 1 (FSSC vs. ALL), we found that FSSC yielded statistically significant greater improvements in CoP SA (*p*<0.0001, *d*=1.55) and CoP V (*p*=0.0004, *d*=1.19). For contrast 2 (FSSC vs. DYM+ISOM), FSSC yielded statistically significant greater improvements in both CoP SA and CoP V (both, *p*<0.0001, *d*=2.03 and 1.45, respectively). Finally, for contrast 3 (FSSC vs. SSSC), FSSC yielded statistically significant greater improvements in CoP SA (*p*=0.04, *d*=0.65), but not in CoP V (*p*=0.14, d=0.47).

**Table 4:** Contrast analyses of conditions for the applied balance test.

		CoP SA				CoP V			
		*p*	Mean diff	T	SE	*p*	Mean diff	T	SE
FSSC	ALL	<0.0001*	490.63	4.92	99.78	0.0004*	13.67	3.76	3.63
FSSC	DYN+ISOM	<0.0001*	608.92	6.43	94.68	<0.0001*	17.19	4.61	3.73
FSSC	SSSC	0.04*	254.05	2.08	122.2	0.14	6.63	1.49	4.44

All differences are manifest from the grand means of the specified contrast(s). DYN, dynamic strength exercise; ISOM, isometric strength exercise; SSSC, slow stretch-shortening cycle exercise; FSSC, fast stretch-shortening cycle exercise, CoP SA, center of pressure surface area; CoP V, center of pressure velocity, p, value of p; Mean diff: mean difference; T, t-value; SE, standard error. *Denotes statistically significant difference.

## Discussion

This study was an investigation of the effects of four strength exercise protocols, involving two plyometric protocols, in fast and slow stretch-shortening cycle (FSSC and SSSC), and two strength exercise protocols, including isometric (ISOM) and dynamic contractions (DYN) on subsequent balance performance in young male and female weightlifters. Based on similar studies (Twist et al., 2008; Kibele et al., 2014; Lauber et al., 2021) and with regard to our research hypothesis, the main finding was that FSSC would show the largest benefits as the FSSC tasks are highly dynamic compared with SSSC, DYN, and ISOM. Our results support the hypothesis that FSSC vs. All (DYN, ISOM, and SSSC) is in favor of FSSC for both outcome parameters (i.e., CoP SA and CoP V). Similarly, for FSSC vs. DYN and ISOM, FSSC yielded significantly greater improvements in both CoP SA and CoP V. Finally, FSSC yielded significantly greater improvements than SSSC only for CoP SA but not for CoP V. Therefore, it is likely that youth weightlifters responded positively to a single bout of FSSC exercise, which consequently improved their balance capacities more than when performing slow plyometric, dynamic, and isometric strength exercises.

The present results indicate a positive acute effect of FSSC on the participants’ balance performance compared to SSSC, DYN, and ISOM in youth weightlifters. Using fast stretch-shortening lengthening, the dynamic nature of the exercises would place a training stress on balance or equilibrium. Furthermore, plyometric exercise can significantly improve neuromuscular control by promoting anticipatory postural adjustments (Gantchev and Dimitrova, 1996). The anticipatory postural adjustments have been shown in peripheral joints. During FSSC exercises, repeated exposure to balance and stability challenges results in proactive, or feed forward adjustments that would activate appropriate muscles prior to landing (Marigold and Patla, 2002; Paillard et al., 2005). Hence, we can speculate that the sensitivity of afferent feedback pathway can be more improved with fast stretch-shortening exercises (Borghuis et al., 2008). Of note, FSSC is a dynamic form of resistance training involving a fast stretch-shortening action [i.e., drop jump (greater base of support can provide less balance challenges); Komi et al., 1987; Kibele et al., 2014], and thus, the FSSC-specific benefits on subsequent balance performance were more pronounced.

Our results implicate that there was a positive influence on balance performance when a FSSC protocol was undertaken compared to DYN and ISOM strength protocols in youth weightlifters. The probable explanation for such a result can be found in physiological characteristics of both types of strength exercises (i.e., DYN and ISOM). In brief, while high-intensity power training (i.e., FSSC exercises) is prevalent in weightlifting training, we conceptualized that strength exercises were less technically demanding compared to the plyometric exercises (Everett, 2016). However, it should be acknowledged that metabolically, this may not be the case. Furthermore, FSSC exercises were performed under less stable conditions with high-speed, dynamic contractions performed within a more limited base of support or with the center of gravity being moved outside the base of support (Taube et al., 2008; Hammami et al., 2016; Lauber et al., 2021), which would be affected to a much greater extent by balance performance than dynamic and isometric strength exercises. Therefore, it is possible that both DYN and ISOM exercises did not sufficiently challenge the study participants’ balance.

Finally, the results of the present study demonstrated that FSSC yielded in greater improvements than SSSC exercises in enhancing subsequent balance performance (i.e., CoP SA) in youth weightlifters. In explaining these results, we must mention that we studied well-trained youth weightlifters, who frequently performed dynamic type of plyometric exercises in their training routines. More importantly, the SSSC protocol comprised exercises that were undertaken with a much lower intensity and volume in comparison with loads regularly applied throughout weightlifting training.

Furthermore, it is important to note that weightlifting athletes exhibit better balance than the average population due to the dynamic nature of their sports rather than better balance *via* specific balance training programs (Vuillerme et al., 2001; Nagy et al., 2004). Hence, since plyometric exercises can provide a spectrum of balance challenges, specific balance exercises may not be needed for all individuals. Thus, the observed findings indicate that adaptive processes, related to both fast and slow stretch-shortening exercises, represent drastically different muscle action patterns, affecting acute balance performance enhancement in young weightlifters.

This study is not without limitations. First, we examined a sample of youth weightlifters. Therefore, the results of this study are specific to the population under investigation. Second, we evaluated the effects of four different strengthening protocols on subsequent balance performance. However, we did not study the acute effects of strength and plyometric exercises on other fitness components that are relevant for weightlifting performance. Given that the acute effects of strength exercises are transient, we had to focus on selected outcome measures (i.e., balance) and could not take additional outcomes into consideration. Future studies should, therefore, examine the acute effects of strength exercises on other fitness components, such as speed. Third, balance was not tested under sport-specific conditions in this study which may have prevented to observe larger effects. Future studies should include balance tests during the performance of weightlifting exercises. Athletes could for instance stand on a force plate while performing the snatch, and clean and jerk. Fourth, we did not include a control condition in the current study which is why the outcomes have to be interpreted with caution. Finally, we were unable to estimate the level of fatigue after the application of the strength and plyometric exercises which could have mitigated the observed balance effects. Future studies could assess ratings of perceived exertion a few minutes after the application of the strength and plyometric exercises to find out whether fatigue may have mitigated the observed balance effects.

## Conclusion

Previous prior studies (Twist et al., 2008; Kibele et al., 2014; Lauber et al., 2021) suggested that plyometric exercises best mimic the subsequent balance performance. The outcomes of the present study confirm the hypotheses-driven study approach. In this context, findings from this study showed positive effects of FSSC exercises/protocols on subsequent balance performance in youth weightlifters compared to SSSC and both dynamic and isometric strength exercises. It is important to note that balance performance in the present study was not tested under sport-specific conditions. Therefore, future studies should include balance tests during the performance of weightlifting exercises. Athletes could for instance stand on a force plate while performing the snatch, and clean and jerk. Furthermore, as balance in weightlifting is directly related to both competitive success and risk of injury (i.e., better balance may serve as protective factor against injury occurrence), these findings should be translated into regular weightlifting training to benefit competitive success and injury risk management. Coaches should therefore be mindful of this finding when working with youth weightlifters in power/strength training settings.

From practical applications, the positive acute effects of fast plyometric exercises on balance performance were more evident than slow plyometric and both dynamic and isometric strength exercises. Therefore, a FSSC exercise protocol (e.g., drop jump), including a small number of sets and repetitions (i.e., 2 to 3, from a 20-cm-high platform) may be suggested as the most valuable pre-workout (pre-training) protocol than SSSC exercises, DYN and ISOM exercises, aimed to the accentuation of balance capacities in prepubertal weightlifters. In addition, while SSSC and ISOM exercises did not result in positive changes of balance performance, the applicability of this type of exercise needs to be further evaluated. It should also be highlighted that, because of the relatively slower dynamic nature of this exercise, SSSC and ISOM exercises may be suggested as a more appropriate strengthening method for less experienced weightlifters.

From a practical or coaches’ point of view, SSSC and ISOM exercises should be avoided as a pre-workout regimen for more experienced athletes. Furthermore, as the competitiveness of sport is reaching into younger ages, coaches and young athletes are seeking training advantages. Because coordination, balance, and power are underdeveloped in youth (Hammami et al., 2021), training programs implementing fast stretch-shortening cycle exercises can accelerate positive changes leading to competitive advantages. The results of this study imply that the FSSC exercises should be introduced to prepubertal weightlifters if the goal is to enhance balance performance which is essential for weightlifting success.

## Data Availability Statement

The raw data supporting the conclusions of this article will be made available by the authors, without undue reservation.

## Ethics Statement

The studies was reviewed and approved by the National Centre of Medicine and Science of Sports, Tunis (CNMSS-LR09SEP01). Written informed consent to participate in this study was provided by the participants’ legal guardian/next of kin.

## Author Contributions

HW, RH, HR, and MS participated in the conception and design of the study. HW, RH, MS, WS, and HR were responsible for testing. HW, MS, CC, UG, DS, GG, and MD were responsible for data collection and statistical analysis. HW, RH, MS, CC, WS, MD, DS, UG, GG, and HR were responsible for the writing and finalization of the manuscript. All authors contributed to the manuscript and approved the submitted version.

## Funding

The authors acknowledge the support of the Deutsche Forschungsgemeinschaft (DFG) and Open Access Publishing Fund of the University of Potsdam, Germany.

## Conflict of Interest

The authors declare that the research was conducted in the absence of any commercial or financial relationships that could be construed as a potential conflict of interest.

## Publisher’s Note

All claims expressed in this article are solely those of the authors and do not necessarily represent those of their affiliated organizations, or those of the publisher, the editors and the reviewers. Any product that may be evaluated in this article, or claim that may be made by its manufacturer, is not guaranteed or endorsed by the publisher.

## References

[ref1] Anderson K. Behm D. G. (2005). The impact of instability resistance training on balance and stability. Sports Med. 35, 43–53. doi: 10.2165/00007256-200535010-00004, PMID: 15651912

[ref2] Asadi A. de Villarreal E. S. Arazi H. (2015). The effects of plyometric type neuromuscular training on postural control performance of male team basketball players. J. Strength Cond. Res. 29, 1870–1875. doi: 10.1519/JSC.0000000000000832, PMID: 25563677

[ref3] Behm D. G. Sale D. G. (1993). Velocity specificity of resistance training. Sports Med. 15, 374–388. doi: 10.2165/00007256-199315060-00003, PMID: 8341872

[ref4] Bobbert M. F. Huijing P. A. van Ingen Schenau G. J. (1987). Drop jumping. I. The influence of jumping technique on the biomechanics of jumping. Med. Sci. Sports Exerc. 19, 332–338. PMID: 3657481

[ref5] Borghuis J. Hof A. L. Lemmink K. A. (2008). The importance of sensory-motor control in providing core stability: implications for measurement and training. Sports Med. 38, 893–916. doi: 10.2165/00007256-200838110-00002, PMID: 18937521

[ref6] Bryanton M. A. Bilodeau M. (2018). The effect of vision and surface compliance on balance in untrained and strength athletes. J. Mot. Behav. 51, 75–82. doi: 10.1080/00222895.2017, PMID: 29377776

[ref7] Caron O. Gélat T. Rougier P. Blanchi J.-P. (2000). A comparative analysis of the center of gravity and center of pressure trajectory path lengths in standing posture: an estimation of active stiffness. J. Appl. Biomech. 16, 234–247. doi: 10.1123/jab.16.3.234, PMID: 11757569

[ref8] Chaouachi A. Hammami R. Kaabi S. Chamari K. Drinkwater E. J. Behm D. G. (2014). Olympic weightlifting and plyometric training with children provides similar or greater performance improvements than traditional resistance training. J. Strength Cond. 28, 1483–1496. doi: 10.1519/JSC.0000000000000305 24172724

[ref42] Deurenberg P. Pieters J. J. Hautvast J. G. (1990). The assessment of the body fat percentage by skinfold thickness measurements in childhood and young adolescence. Br. J. Nutr. 63, 293–303. doi: 10.1079/bjn19900116 2334665

[ref9] Everett G. (2016). Olympic Weightlifting: A Complete Guide for Athletes & Coaches; Catalyst. Sunnyvale: Catalyst Athletics, 628.

[ref10] Faigenbaum A. (2009). Youth Resistance Training. Colorado Springs: NSCA Hot Topic Series.

[ref11] Faigenbaum A. McFarland J. Johnson L. Kang J. Bloom J. Ratamess N. A.. (2007). Preliminary evaluation of an afterschool resistance training program. Percept. Mot. Skills 104, 407–415. doi: 10.2466/pms.104.2.407-415, PMID: 17566430

[ref44] Faigenbaum A. D. McFarland J. E. Schwerdtman J. A. Ratamess N. A. Kang J. Hoffman J. R. (2006). Dynamic warm-up protocols, with and without a weighted vest, and fitness performance in high school female athletes. J. Athl. Train. 41, 357–363. 17273458 PMC1748418

[ref40] Flanagan E. P. Ebben W. P. Jensen R. L. (2008). Reliability of the reactive strength index and time to stabilization during depth jumps. J. Strength Cond. Res. 22, 1677–1682. doi: 10.1519/JSC.0b013e318182034b 18714215

[ref12] Gantchev G. N. Dimitrova D. M. (1996). Anticipatory postural adjustments associated with arm movements during balancing on unstable support surface. Int. J. Psychophysiol. 22, 117–122. doi: 10.1016/0167-8760(96)00016-5 8799774

[ref13] Granacher U. Muehlbauer T. Doerflinger B. Strohmeier R. Gollhofer A. (2011). Promoting strength and balance in adolescents during physical education: effects of a fast-term resistance training. J. Strength Cond. Res. 25, 940–949. doi: 10.1519/JSC.0b013e3181c7bb1e, PMID: 20661162

[ref14] Haans A. (2008). Contrast analysis: A tutorial. Pract. Assess. Res. Eval. 23, 1–21. doi: 10.7275/7dey-zd62

[ref15] Hammami R. Chaabene H. Kharrat F. Werfelli H. Duncan M. Rebai H.. (2021). Acute effects of different balance exercise types on selected measures of physical fitness in youth female volleyball players. BMC Sports Sci. Med. Rehabil. 13:29. doi: 10.1186/s13102-021-00249-5 33743814 PMC7981889

[ref16] Hammami R. Duncan M. J. Nebigh A. Werfelli H. Rebai H. (2020). The effects of 6 weeks eccentric training on speed, dynamic balance, muscle strength, power, and lower limb asymmetry in prepubescent weightlifters. J. Strength Cond. doi: 10.1519/JSC.0000000000003598 32483061

[ref17] Hammami R. Granacher U. Maklouf I. Behm D. G. Chaouachi A. (2016). Sequencing effects of balance and plyometric training on physical performance in youth soccer athletes. J. Strength Cond. Res. 30, 3278–3289. doi: 10.1519/jsc.0000000000001425 27144955

[ref18] Haycraft J. A. Gastin P. B. Robertson S. (2016). The acute effect of maximal voluntary isometric contraction pull on start gate performance of snowboard and ski cross athletes. Int. J. Sports Sci Coaching 11, 721–727. doi: 10.1177/1747954116667110

[ref19] Hervé A. Williams L. J. (2010). Contrast analysis. Encycl. Res Des. 1–14.

[ref45] Hopkins W. G. (2002). A scale of magnitudes for effect statistics. Sports Sci. 5, 1–7.

[ref46] Hopkins W. G. (2005). Competitive performance of elite track-and-field athletes: variability and smallest worthwhile enhancements. Sports Sci. 9, 17–20. doi: 10.1123/ssj.20.1.17

[ref20] Kang S. H. Kim C. W. Kim Y. I. Kim K. B. Lee S. S. Shin K. O.. (2013). Alterations of muscular strength and left and right limb balance in weightlifters after an 8-week balance-training program. J. Phys. Ther. Sci. 25, 895–900. doi: 10.1589/jpts.25.895, PMID: 24259879 PMC3820381

[ref21] Kibele A. Classen C. Muehlbauer T. Granacher U. Behm D. G. (2014). Metastability in plyometric training on unstable surfaces: a pilot study. BMC Sports Sci. Med. Rehabil. 6:30. doi: 10.1186/2052-1847-6-30 25089202 PMC4118276

[ref22] Komi P. V. Bosco C. (1978). Utilization of stored elastic energy in leg extensor muscles by men and women. Med. Sci. Sports 10, 261–265. PMID: 750844

[ref41] Komi P. V. Salonen M. Järvinen M. Kokko O. (1987). In vivo registration of Achilles tendon forces in man. Int. J. Sports Med. 8, S3–S8. doi: 10.1055/s-2008-1025697 3583517

[ref23] Lauber B. Gollhofer A. Taube W. (2021). What to train first: balance or explosive strength? Impact on performance and intracortical inhibition. Scand. J. Med. Sci. Sports 31, 1301–1312. doi: 10.1111/sms.13939, PMID: 33606302

[ref24] Marigold D. S. Patla A. E. (2002). Strategies for dynamic stability during locomotion on a slippery surface: effects of prior experience and knowledge. J. Neurophysiol. 88, 339–353. doi: 10.1152/jn.00691.2001, PMID: 12091559

[ref43] Moore S. A. McKay H. A. Macdonald H. Nettlefold L. Baxter-Jones A. D. G. Cameron N.. (2015). Enhancing a somatic maturity prediction model. Med. Sci. Sports Exerc. 47, 1755–1764. doi: 10.1249/MSS.0000000000000588 25423445

[ref25] Nagy E. Toth K. Janositz G. Kovacs G. Feher-Kiss A. Horvath G.. (2004). Postural control in athletes participating in an ironman triathlon. Eur. J. Appl. Physiol. 92, 407–413. doi: 10.1007/s00421-004-1157-7, PMID: 15205962

[ref26] Paillard L. C. Soulat J. M. Montoya R. Costes-Salon M. C. Duput P. (2005). Shortterm effects of electrical stimulation superimposed on muscular voluntary contraction in postural control in elderly women. J. Strength Cond. Res. 19, 640–646. doi: 10.1519/15354.1, PMID: 16095419

[ref27] Pancar Z. Bozdal Ö. Biçer M. Akcan F. (2017). Acute effect of anaerobic exercise on dynamic balance of sedentary young boys. Eur. J. Phys. Educ. Sport. Sci. 3, 229–237. doi: 10.5281/zenodo/1098529

[ref39] Pearson S. J. Young A. Macaluso A. Devito G. Nimmo M. A. Cobboldet M.. (2002). Muscle function in elite master weightlifters. Med. Sci. Sports Exerc. 34, 1199–1206. doi: 10.1097/00005768-200207000-00023 12131263

[ref28] Riemann B. L. Mercado M. Erickson K. Grosicki G. J. (2020). Comparison of balance performance between masters Olympic weightlifters and runners. Scand. J. Med. Sci. Sports 30, 1586–1593. doi: 10.1111/sms.13729, PMID: 32474974

[ref29] Rosnow R. L. Rosenthal R. (1995). “Some things you learn aren’t so”: Cohen’s paradox, Asch’s paradigm, and the interpretation of interaction. Psychol. Sci. 6, 3–9. doi: 10.1111/j.1467-9280.1995.tb00297.x

[ref30] Rosnow R. L. Rosenthal R. (1996). Contrasts and interaction redux: five easy pieces. Psychol. Sci. 7, 253–257. doi: 10.1111/j.1467-9280.1996.tb00369.x

[ref31] Sander A. Keiner M. Wirth K. Schmidtbleicher D. (2013). Influence of a 2-year strength training programme on power performance in elite youth soccer players. Eur. J. Sport Sci. 13, 445–451. doi: 10.1080/17461391.2012.742572, PMID: 24050460

[ref32] Schubert P. Kirchner M. (2014). Ellipse area calculations and their applicability in posturography. Gait Posture 39, 518–522. doi: 10.1016/j.gaitpost.2013.09.001, PMID: 24091249

[ref33] Stone M. H. Sands W. A. Pierce K. C. Carlock J. Cardinale M. Newton R. U.. (2005). Relationship of maximum strength to weightlifting performance. J. Med. Sci. Sports Exerc. 37, 1037–1043. PMID: 15947731

[ref34] Szafraniec R. Bartkowski J. Kawczyński A. (2020). Effects of fast-term core stability training on dynamic balance and trunk muscle endurance in novice Olympic weightlifters. J. Human Kinetics. 74:43. doi: 10.2478/hukin-2020-0012, PMID: 33312274 PMC7706638

[ref35] Taube W. Gruber M. Gollhofer A. (2008). Spinal and supraspinal adaptations associated with balance training and their functional relevance. Acta Physiol. 193, 101–116. doi: 10.1111/j.1748-1716.2008.01850.x 18346210

[ref36] Taube W. Schubert M. Gruber M. Beck S. Faist M. Gollhofer A.. (2006). Direct corticospinal pathways contribute to neuromuscular control of perturbed stance. J. Appl. Physiol. 101, 420–429. doi: 10.1152/japplphysiol.01447.2005, PMID: 16601305

[ref37] Twist C. Gleeson N. Eston R. (2008). The effects of plyometric exercise on unilateral balance performance. J. Sports Sci. 26, 1073–1080. doi: 10.1080/02640410801930168, PMID: 18608837

[ref38] Vuillerme N. Teasdale N. Nougier V. (2001). The effect of expertise in gymnastics on proprioceptive sensory integration in human subjects. Neurosci. Lett. 311, 73–76. doi: 10.1016/S0304-3940(01)02147-4, PMID: 11567781

